# Generation of 3D reference dosimetric datasets towards adoption of model‐based dose calculations for permanent implant prostate brachytherapy

**DOI:** 10.1002/mp.70309

**Published:** 2026-02-10

**Authors:** Fatemeh Akbari, Vasiliki Peppa, Samuel Ouellet, Narjes Moghadam, Sandra Oliver, Vicent Gimenez‐Alventosa, Luc Beaulieu, Javier Vijande, Rowan M. Thomson

**Affiliations:** ^1^ Carleton Laboratory for Radiotherapy Physics Physics Department Carleton University Ottawa Ontario Canada; ^2^ Medical Physics Laboratory Medical School National and Kapodistrian University of Athens Athens Greece; ^3^ Département de physique de génie physique et d'optique et Centre de recherche sur le cancer Université Laval Québec Canada; ^4^ Service de radio‐oncologie et Axe Oncologie du CRCHU de Québec CHU de Québec‐Université Laval Quebec QC Canada; ^5^ Instituto de Seguridad Industrial Radiofísica y Medioambiental (ISIRYM) Universitat Politècnica de València València Spain; ^6^ Departamento de Física Atómica Molecular y Nuclear Universitat de Valencia (UV) Burjassot Spain; ^7^ Instituto de Física Corpuscular IFIC (UV‐CSIC) Burjassot Spain

**Keywords:** implant prostate brachytherapy, model‐based calculation, Monte Carlo

## Abstract

**Purpose:**

This work aims to create and validate a comprehensive set of test cases and 3D reference dosimetric datasets for model‐based dose calculation algorithms (MBDCAs) in permanent implant prostate brachytherapy. These test cases address the limits of the standard TG‐43 formalism towards improving dose evaluations in prostate cancer treatment, in accordance with the recommendations of the joint Task Group 186 of the American Association of Physicists in Medicine (AAPM), the European Society for Radiotherapy and Oncology (ESTRO), and the Australasian Brachytherapy Group (ABG).

**Acquisition and Validation Methods:**

Six test scenarios using the I‐125 OncoSeed 6711 were simulated with three Monte Carlo codes: EGSnrc application egs_brachy with eb_gui, MCNP with BrachyGuide, and PenRed. The test cases range from basic, e.g., single seed in water, to more complex configurations, e.g., 58 seeds in a virtual patient model for prostate treatment. The virtual model was developed from anonymized CT‐based images, representing a real patient with and without calcifications. Dose comparisons were performed using both local and global metrics, with eb_gui as the reference for validation. For all test cases, the local agreement of Monte Carlo codes was within ∼3.0% and the global agreement was 0.01%.

**Data Format and Usage Notes:**

The dataset, including Monte Carlo input files and other required information, is available online at https://doi.org/10.5281/zenodo.15282647.

**Potential Applications:**

The developed test cases provide an essential resource for the development, validation, and commissioning of MBDCAs. They have the potential to improve dosimetric accuracy in complex prostate implants while also providing a platform for future brachytherapy research.

## 1 INTRODUCTION

Advancements in medical technology have significantly transformed cancer treatment, particularly in the field of prostate brachytherapy. Permanent implant prostate brachytherapy (PIPB), a technique in which radioactive seeds are precisely implanted directly into the prostate, has emerged as a highly effective method for treating localized prostate cancer.^1^, ^2^, ^3^ Despite its effectiveness, the precision of dose delivery and the accuracy of dose calculations are critical factors in optimizing treatment outcomes and minimizing potential side effects.

Currently, clinical dose estimations for brachytherapy rely on the formalism developed by the American Association of Physicists in Medicine (AAPM) Task Group 43 (TG‐43).^4^ This approach generates dosimetric data based on a single source within a homogeneous water phantom and assumes negligible interseed, non‐water tissue, and implant‐ or anatomy‐ related geometry effects. However, this simplification can lead to inaccuracies in dose distribution data, particularly for low‐energy brachytherapy sources where the impact of these parameters is more pronounced due to the predominance of the photoelectric effect. Recognizing these limitations, TG‐186^5^ recommends employing model‐based dose calculation algorithms (MBDCAs) as an alternative to TG‐43. MBDCAs incorporate patient‐specific scattering conditions, heterogeneities, and radiation interactions in the source and applicator materials, utilizing material composition and density information derived from Computed Tomography (CT) images. The enhanced performance of MBDCAs has been shown in the literature for radiotherapy of cervix, breast, and prostate cancers.^6^, ^7^, ^8^, ^9^, ^10^, ^11^ More recent investigations have also explored the use of cone‐beam CT for TG‐186‐based dose calculations and the application of 3D‐printed, patient‐individual moulds,^12^, ^13^ further highlighting the versatility and expanding clinical relevance of MBDCAs.

In typical PIPB implants, which contain 40–100 seeds in close proximity, seed‐to‐seed interference can substantially alter dose distributions. This variation depends on the specific model used and the number of seeds in the implant.^14^, ^15^, ^16^ For instance, the dose distribution around a single I‐125 seed model 6711^17^ can be perturbed by up to 10% due to the presence of nearby seeds.^18^, ^19^ Additionally, dose coverage calculated for the 150% and 200% isodose lines was found to be 29% and 136% higher, respectively, compared to TG‐43 computations.^20^ Patient‐specific Monte Carlo (MC) simulations have shown a 5.9% ± 1.6% decrease in D_90_ for the prostate and a 4.4% ± 1.8% decrease in D_5_ for the urethra compared to TG‐43 formalism.^21^ Furthermore, calcifications can cause deviations in dose metrics that differ by as much as 58%, depending on their volume.^22^

To address these challenges, a joint working group of the AAPM, the European Society for Radiotherapy and Oncology (ESTRO), the American Brachytherapy Society (ABS), and the Australasian Brachytherapy Group (ABG) is creating and benchmarking test cases to compare MBDCAs with traditional TG‐43 calculations. Test cases for interstitial high dose rate (HDR) breast^23^ and low dose rate (LDR) COMS eye plaque brachytherapy,^24^ created by the Working Group on MBDCAs in Brachytherapy (WGMBDCA), are accessible at the Joint AAPM/IROC Houston model‐based dose calculations database.^25^ Nevertheless, there are currently no dosimetric datasets for PIPB.

This work presents the creation of a comprehensive suite of test cases and 3D reference dosimetric datasets specifically designed for MBDCA validation, following the guidelines provided by AAPM‐ESTRO‐ABS‐ABG working group and TG‐186.^26^ This new framework aims to improve personalized treatment planning by integrating high‐resolution imaging with advanced computational models. The proposed series of Monte Carlo based test cases ranging from single‐source models in water to multiple‐seed configurations within realistic settings, are intended to bridge the gap between conventional dosimetric methods and the evolving requirements of customized cancer therapy. While commercially approved MBDCAs are available for temporary HDR prostate brachytherapy, no commercial MBDCA currently exists for permanent low‐dose‐rate (LDR) prostate brachytherapy. Therefore, these test cases will be crucial for developing, validating, and commissioning algorithms in permanent LDR prostate brachytherapy, ultimately enhancing dosimetric precision in PIPB and offering a valuable resource for the research community.

This study represents an independent investigation by a collaboration that includes members of the WGMBDCA working group and should not be construed as an official recommendation by the societies mentioned.

## 2 ACQUISITION AND VALIDATION METHODS

This study includes six test cases, detailed in Table 1, all utilizing I‐125 OncoSeed 6711. Simulations were conducted using three Monte Carlo codes: EGSnrc application egs_brachy with eb_gui (egs_brachy graphical user interface),^27^ MCNP with BrachyGuide,^28^ and PenRed.^29^ The collection of test cases ranges from simple scenarios (such as a single seed in water) to more complex ones (e.g., multiple seeds in a full‐ tissue phantom) which are further explored in Section 2.1.1. These test cases establish a structured approach for assessing model‐based dose calculations specifically for I‐125 prostate LDR treatment.

**TABLE 1:** Overview of the studied test cases.

Test case number	Description	Seed configuration	Phantom
1	Single seed in water (TG‐43)	Single seed	Water
2	Single seed in phantom	Single seed	CT‐based full tissue phantom
3	Multiseed, no interseed effects (TG‐43)	58 seeds	Water
4	Multiseed, with interseed effects	58 seeds	Water
5	Virtual patient without calcifications	58 seeds	CT‐based full tissue phantom, without calcifications
6	Virtual patient with calcifications	58 seeds	CT‐based full tissue phantom, with calcifications

### 2.1 Methods

#### 2.1.1 Test cases

The well‐characterized I‐125 model 6711 seed^30^, ^31^ was used for each test case to ensure the study results were independent of specific vendors. The initial photon emission spectra were taken from the National Nuclear Data Center NuDat3.0, with the minor 3.77 keV peak excluded from the probability distribution function of the I‐125 emission spectrum. Table 2 shows the photon spectrum used in this work.

**TABLE 2:** I‐125 spectrum used in this work.

Energy (keV)	% Intensity
27.202	39.6
27.472	73.1
30.944	6.74
30.995	13.0
31.704	3.75
35.4925	6.68

**FIGURE 1:**
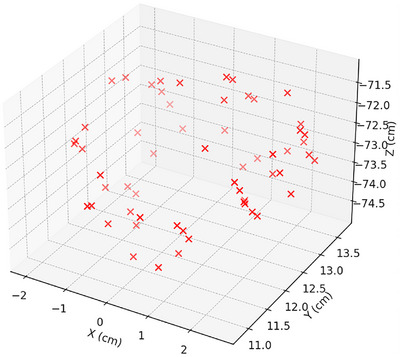
Three‐dimensional scatter plot of the I‐125 seed positions in the phantom coordinate system. Each point represents the (*x*, *y*, *z*) location of a seed used in the test cases 3–6.

The first two test cases simulated a single seed centered at coordinates (0.735 cm, 12.9 cm, −71.2 cm) with its long axis directed along the Z‐axis. Seed positions for other cases are provided in Table 3, and their 3D placements are illustrated in Figure 1.

**TABLE 3:** Seed center coordinates for multiseed test cases (test cases 3–6); seeds are oriented with their long axis in the *Z*‐direction.

Seed number	*X* (cm)	*Y* (cm)	*Z* (cm)	Seed number	*X* (cm)	*Y* (cm)	*Z* (cm)
1	−1.344	13.192	−73.940	30	2.590	13.235	−73.000
2	−1.542	12.139	−74.003	31	2.071	12.122	−73.500
3	−0.727	11.392	−74.034	32	2.034	11.936	−73.100
4	1.183	12.941	−74.188	33	2.034	11.676	−72.400
5	−0.712	11.936	−74.200	34	2.553	13.109	−72.765
6	−0.640	12.005	−74.587	35	2.482	12.373	−72.622
7	−1.343	12.715	−74.600	36	−1.937	11.973	−73.000
8	−1.429	12.591	−74.330	37	−1.949	12.066	−72.532
9	0.651	11.748	−74.668	38	2.0234	11.827	−72.730
10	0.693	11.191	−74.500	39	−1.863	11.751	−72.600
11	−0.226	11.504	−74.815	40	2.049	13.57	−72.966
12	0.735	12.901	−71.200	41	−2.011	11.855	−72.795
13	1.877	13.265	−71.553	42	−0.848	11.349	−72.815
14	0.709	13.098	−71.419	43	−0.629	10.816	−73.062
15	−0.203	12.697	−71.441	44	0.062	13.628	−73.287
16	−0.956	12.988	−71.819	45	1.699	13.527	−73.262
17	0.602	12.994	−71.872	46	2.652	12.496	−73.196
18	0.846	13.383	−72.000	47	1.952	12.058	−73.280
19	−1.837	12.989	−71.969	48	0.499	10.949	−73.100
20	2.516	13.012	−72.200	49	−1.729	13.172	−73.493
21	−2.039	12.839	−71.992	50	1.765	13.035	−73.419
22	2.513	12.876	−71.987	51	1.069	11.268	−73.400
23	2.485	12.161	−71.994	52	−0.841	11.117	−73.374
24	0.856	13.536	−72.196	53	−0.963	13.582	−73.500
25	−1.417	13.717	−73.100	54	2.140	12.183	−73.635
26	−1.492	13.309	−72.300	55	1.069	11.565	−74.000
27	0.974	12.082	−72.229	56	1.084	11.404	−73.646
28	1.997	13.532	−72.500	57	0.861	13.658	−73.807
29	−1.417	13.495	−72.600	58	0.690	13.443	−73.899

An anonymized Digital Imaging and Communications in Medicine (DICOM) dataset, which included CT images, RTplan and RTstructure was utilized to create a complex, heterogenous voxelized phantom. This virtual model was constructed from the post‐implant CT images of a real patient, ensuring the model's clinical relevance. The phantom had dimensions of (19 × 18.99 × 19.8) cm^3^ and consisted of 512 × 512 × 99 voxels in the *X*, *Y*, and *Z* directions, respectively. The dose scoring grid had the same resolution as the original patient imaging data with voxel sizes of 0.371 mm × 0.371 mm × 2 mm. The prostate model incorporated various anatomical structures, including bladder, rectum, urethra, target (prostate), and calcifications. Each structure was assigned a nominal Hounsfield unit (HU), and a default HU to density table was used to compute the corresponding nominal density for each region of interest. In accordance with the recommendations of TG‑186—which acknowledge the challenge of assigning voxel‐by‐voxel material composition from CT and allow the use of structure‐based uniform densities for commissioning and benchmarking—a uniform density was assigned to each anatomical structure in this reference dataset. This simplified the debugging process and minimized potential sources of error and facilitated a more straightforward identification and resolution of any issues, as it removes the complexities that arise from varying material densities. Although this approximation may not fully reflect the true heterogeneity of calcifications and high‑density materials, it provides a reproducible and controlled basis for cross‐algorithm evaluation, consistent with practices used in other published test cases.^23^

**FIGURE 2:**
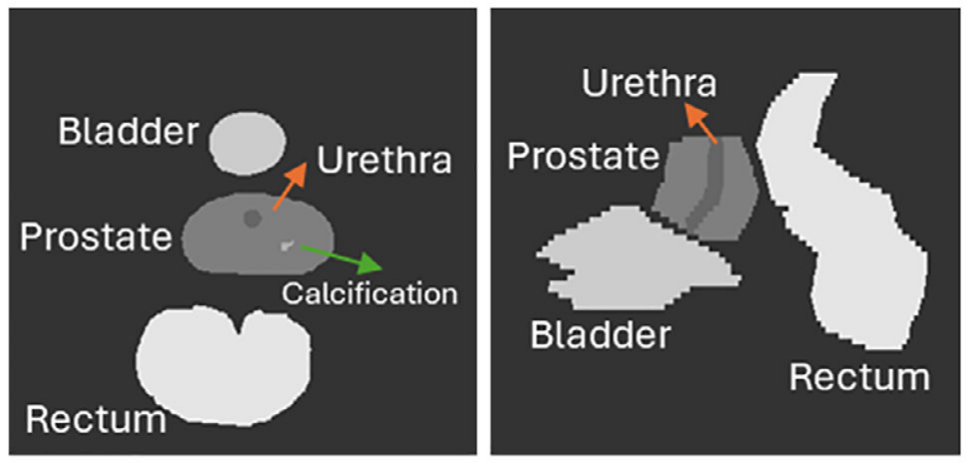
An image showing the patient phantom model used in this work. (a) *X*–*Y* cross‐section at *Z* = −72.1 cm. (b) *Y*–*Z* cross‐section at *X* = 0.16 cm.

An image of the phantom model is presented in Figure 2. The elemental composition and mass density of tissues used in tissue assignment scheme are summarized in Table 4.

**TABLE 4:** Elemental composition and mass density of tissues used in tissue assignment scheme.^32^

		Elemental composition (Mass %)					
Region	Tissue assignment	H	C	N	O	Elements with *Z* > 8	Density(g/cm^3^)
Target	Prostate	10.5	8.9	2.5	77.4	Na (0.2), P (0.1), S (0.2), K (0.2)	1.04
	Calcification	0.3	1.6	0.5	40.7	P (18.7), Ca (38.2)	3.06
Bladder	Urinary Bladder (Empty)	10.5	9.6	2.6	76.1	Na (0.2), P (0.2), S (0.2), Cl (0.3), K (0.3)	1.04
Rectum	Rectum	6.3	12.1	2.2	79.0	Na (0.01), P (0.1), Cl (0.1), K (0.1)	0.75
Urethra	Prostate	10.5	8.9	2.5	77.4	Na (0.2), P (0.1), S (0.2), K (0.2)	1.04
Soft tissue	Male soft tissue	10.5	25.6	2.7	60.2	Na (0.1), P (0.2), S (0.3), Cl (0.2), K (0.2)	1.00

Test cases evolved from a single‐seed scenario to a more complex representation of a real patient case, progressing from an initial water medium to a more realistic setting involving full tissue assignment. The first test case simulated a single seed in the phantom where all voxels were replaced with water to replicate the conditions outlined in the TG‐43 protocol. This simplified scenario allowed for an initial validation of the model under uniform, homogenous conditions. The second test case modeled a single seed placed within a full virtual patient model, providing a more realistic tissue distribution while still focusing on a single seed. This setup aimed to evaluate the dosimetric impact of tissue heterogeneity on the seed's dose distribution. The third test case involved a scenario with 58 seeds placed in water under TG‐43 reference conditions. To replicate the TG‐43 assumption of independent seed contributions, the simulation was configured such that only one seed was active at a time, effectively neglecting interseed attenuation effects. This allowed for a straightforward comparison of dose distributions without the complexity of seed interactions. The fourth test case was identical to the third but included interseed attenuation, with all 58 seeds modeled simultaneously. This test case sought to account for the attenuation and scatter interactions between seeds, which can influence the overall dose distribution in a real clinical setup. The fifth test case represented a highly realistic scenario, in which 58 seeds were positioned within the CT‐based full‐tissue phantom and interseed effects were taken into account. The sixth test case—the most realistic scenario—was similar to the fifth but included calcifications within the phantom to evaluate their impact on dosimetric comparisons. This allowed for an investigation into how the presence or absence of calcifications might affect the dose distribution and overall treatment planning.

For all test cases, a minimum of 10^11^, 10^10^, and 10^9^ histories were simulated using eb_gui, MCNP, and PenRed, respectively.

#### 2.1.2 Monte Carlo codes

In this study, we utilized the EGSnrc (egs_brachy), MCNP6, and PenRed Monte Carlo codes. Each of these codes estimates the absorbed dose by calculating the collision kerma. The photon fluence spectrum, recorded in the voxels through the tracklength estimator, is converted into collision kerma using the mass energy‐absorption coefficient of the medium being scored. Each code offers a unique set of features and capabilities suited to different aspects of brachytherapy dose calculation.

EGSnrc calculations were performed using eb_gui.^27^, ^33^ The eb_gui is a free, open‐source software tool designed for rapid MC simulations of brachytherapy treatment plans. It utilizes the egs_brachy code, which is specifically developed for brachytherapy dose calculations. The software is available for download at https://github.com/clrp‐code/egs_brachy/. MCNP simulations used MCNP6^34^ with BrachyGuide.^28^ It should be noted that BrachyGuide was used to parse the information from the treatment plans, yet the MCNP input files were manually edited since the software does not support low‐energy brachytherapy sources in its current version. BrachyGuide is available for download at https://mpl‐en.med.uoa.gr/downloads/. The PenRed simulation used PenRed v.1.11.0b^29^ which implements a special type of mesh‐based geometry to enable simulations directly from DICOM images. The software is available for download at https://github.com/PenRed/PenRed.

Information on the MC simulations performed independently is presented in Table 5 following RECORDS (improved Reporting of montE CarlO RaDiation transport Studies).^35^ The calculations with eb_gui were “ab initio”, that is, they did *not* make use of egs_brachy's features that enhance simulation efficiency (particle recycling (of photons emitted from seeds) and/or use of a phase space source) to provide reference dosimetric data and associated statistical uncertainties evaluated using history‐by‐history statistics.^36^ In contrast, both MCNP and PenRed simulations made use of phase space sources and nominally used history‐by‐history statistics for evaluation of uncertainties, however, the computation of statistical uncertainties did not account for correlations between particles from the same history that were initiated at different source locations.

**TABLE 5:** Summary of methods used for Monte Carlo simulations of this work following the TG 268^35^ template.

Item	egs_brachy	MCNP	PenRed
Code	eb_gui (2023‐commit‐c14a205),^27^, ^37^ egs brachy (v2017.10.02),^36^ EGSnrc (v2019a‐15‐g049a881)^38^	MCNP6 v.6.2^34^ BrachyGuide^28^	PenRed v1.11.0b^29^
Validation	Calculations of TG‐43 parameters,^31^ Brachytherapy treatment simulations^36^	^8^, ^28^, ^39^, ^40^, ^41^, ^42^	Brachytherapy treatment simulations^43^
Timing	Single core on AMD Ryzen 9 5900X. 4.20 GHz CPU Case 1,2: ∼ 45 h per 10^10^ histories Case 3–6: ∼ 60 h per 10^10^ histories	Two 6‐core CPUs (24 computational threads) clocked at 2.3 GHz Cases 1,3,4: ∼12 h per 10^10^ histories Case 2: ∼1.5 day per 10^10^ histories Cases 5,6: ∼25 days per 10^10^ histories	Intel(R) Core (TM) i9‐10900K CPU @ 3.70 GHz (20 logical threads) Case 1: 44 h per 10^10^ histories Case 2: 40 h per 10^10^ histories Case 3: 988 h per 10^9^ histories Case 4: 1220 h per 10^9^ histories Case 5: 1323 h per 10^9^ histories Case 6: 2201 h per10^9^ histories
Source description	Model 6711 I‐125 seed^17^	Case 1: Model 6711 I‐125 seed^17^ Cases 2,3,4,5,6: Model 6711 I‐125 seed^29^ represented by a phase space file of photons emerging from the source for 8 × 10^8^ initially emitted photons	Cases 1,2: Model 6711 I‐125^17^ seed Cases 3,4: Model 6711 I‐125 seed^29^ represented by a phase space file of photons emerging from the source for 1 × 10^9^ initially emitted photons (1 × 10^11^ applying splitting factor of 100)
Cross‐sections	XCOM,^44^ mass‐energy absorption data precalculated^36^	EPDL97^45^	Photoelectric from PHOTACS^46^, ^47^ Compton from relativistic impulse approximation^48^ EPDL97^45^
Transport parameters	Photon transport to 1 keV, No electron transport		
Variance reduction	Model photon transport only	–	Splitting particles used for PSF source
Scored quantities	Collision kerma to local medium in voxels using track‐length estimator		
Statistical uncertainties	Type A uncertainty under 0.5% for voxels within the 1 Gy isodose line thresholds in the single‐seed test cases, and under 2% for voxels within the 20 Gy isodose line threshold in the multi‐seed test cases	Type A uncertainty of under 2.2% for voxels within the 1 Gy isodose line threshold in the single‐seed test cases, and under 3.1% for voxels within the 20 Gy isodose line threshold in the multi‐seed test cases	Type A uncertainty under 2% for voxels within the 1 Gy and 20 Gy isodose line thresholds in the single‐seed and multi‐seed test cases, respectively
Statistical methods	History by history calculation of statistical uncertainties		
Post‐processing	Absorbed dose per history was divided by the air‐kerma strength per history factor as calculated by each code. Quantities of interest were calculated using in‐house codes		

For each code, the air‐kerma strength was used for the calculation of absolute dose in Gy. The air‐kerma strength per history factor ($S_{k}^{hist}$) value was determined using the National Institute of Standards and Technology (NIST) WAFAC (Wide Angle Free‐Air Chamber) geometry as $S_{k}^{hist}={\dot{K}}_{\delta }\left(d\right)\times d^{2}\times k_{r^{2}}$, where ${\dot{K}}_{\delta }\left(d\right)$ represents the air kerma per history due to photons with energies greater than 𝛿 = 5 keV in a 2.7 × 2.7 × 0.05 cm^3^ voxel at d = 10 cm from the source, and the factor $k_{r^{2}}$ corrects for inverse‐square effects over the scoring voxel. The correction was calculated as 1.0172.^49^ The results of the calculated $S_{k}^{hist}$ factors are summarized in Table 6. As shown in this table, the $S_{k}^{hist}$ values obtained from the different codes are in good agreement. The observed small variations are expected, as different Monte Carlo codes may use slightly different cross‐section data or methods for handling photon interactions, especially at lower energies.^50^

**TABLE 6:** Air‐kerma strength per history factors as calculated by each MC code.

	S_k_ [×10^−14^ Gy cm^2^/ history]	Ratio to egs_brachy	Statistical uncertainty
egs_brachy	3.7666	1.000	0.03%
MCNP	3.7426	0.994	0.03%
PenRed	3.7603	0.998	0.03%

#### 2.1.3 Dose scoring and comparison metrics

To quantify the differences in dose distributions voxel‐by‐voxel, two 3D dose comparison metrics were used as follows:

(1) $$\Delta D_{LOCAL}\;\left(\%\right)=\frac{D\left(r\right)-D_{ref}\left(r\right)}{D_{ref}\left(r\right)}\;\;\times 100\%$$

(2) $$\Delta D_{GLOBAL}\;\left(\%\right)=\frac{D\left(r\right)-D_{ref}\left(r\right)}{D_{Rx}}\;\times 100\%$$

In these equations, D(r) is the dose within the voxel containing point r determined using one MC and $D_{ref}\left(r\right)$ is the reference dose from eb_gui. The prescription dose value (144 Gy) was selected for $D_{Rx}$ to calculate the global dose difference ratio. eb_gui was selected as the reference MC dataset for all comparisons, primarily due to its relatively lower Type A uncertainty compared to the other MC codes. In our analysis, voxels that overlap with the seeds were excluded to avoid ambiguities in comparing results (e.g., egs_brachy is the only code that can account for the voxel volume excluded in computing doses)—these voxels are a small fraction compared to the total voxel count in the phantom.

### 2.2 Results

#### 2.2.1 Dose comparisons

Results of the comparisons for all test cases are summarized in Table 7. The table provides the ranges of $\Delta D_{GLOBAL}\left(\%\right)$ for all voxels in the phantom, as well as the ranges of $\Delta D_{LOCAL}\left(\%\right)$ that encompass 95% of the voxels. These ranges provide an overview of the agreement between the dose distributions from the different Monte Carlo codes and the reference dose.

**TABLE 7:** Statistical results for the local and global dose difference ratios, with eb_gui results used as $D_{ref}$. The first three data columns give the ranges of $\Delta D_{GLOBAL}\left(\%\right),$ the means of the $\Delta D_{GLOBAL}\left(\%\right)$ distributions, and the position of the peaks in the $\Delta D_{GLOBAL}\left(\%\right)$ distributions. The next three columns give the same data for the $\Delta D_{LOCAL}\left(\%\right)$ distributions.

		$\Delta D_{GLOBAL}\left(\%\right)$			$\Delta D_{LOCAL}\left(\%\right)$		
		Range	Mean	Peak	Range	Mean	Peak
Test case 1	MCNP	−0.01, 0.01	0.00	−0.01	−1.73, −0.12	−0.92	−0.90
	PenRed	−0.02, 0.01	0.00	0.00	−3.07, −1.00	−2.03	−2.10
Test case 2	MCNP	0.01, 0.01	0.00	−0.01	−2.62, 0.89	−0.86	−0.90
	PenRed	−0.01, 0.01	0.00	0.00	−2.93, −1.16	−2.10	−2.40
Test case 3	MCNP	−0.10, 0.12	0.00	0.00	−2.71, 1.99	−0.36	−0.30
	PenRed	−0.09, 0.09	0.00	0.00	−3.14, −0.21	−1.67	−1.10
Test case 4	MCNP	−0.12, 0.12	0.00	0.00	−3.60, 1.82	−0.88	−0.90
	PenRed	−0.12, 0.10	−0.01	0.00	−4.42, −1.16	−2.80	−2.40
Test case 5	MCNP	−0.13, 0.10	−0.01	−0.01	−3.65, 1.40	−1.12	−1.10
	PenRed	−0.13, 0.10	−0.01	0.00	−4.54, −1.57	−3.06	−2.90
Test case 6	MCNP	−0.13, 0.10	−0.01	−0.01	−3.73, 1.55	−1.09	−1.00
	PenRed	−0.13, 0.10	−0.01	0.00	−4.62, −1.56	−3.09	−3.00

To focus on clinically relevant regions, where dose variations are more impactful, statistical results were calculated by excluding low‐dose voxels, which are less significant in clinical decision‐making. Specifically, for test cases 1 and 2, a dose threshold of 1 Gy was applied to exclude low‐dose regions, ensuring that only voxels receiving clinically relevant doses were considered for the calculation of $\Delta D_{LOCAL}\left(\%\right)$. For test cases 3 through 6, a higher threshold of 20 Gy was used, reflecting the higher dose levels typically encountered in the target regions of interest in multi‐seed prostate brachytherapy scenarios. This approach helps to mitigate the influence of regions with minimal therapeutic impact, providing a more relevant comparison of dose distributions in areas that directly affect tumor control and normal tissue sparing.

For all test cases, the mean values of $\Delta D_{LOCAL}\left(\%\right)$ are less than 1.12% for MCNP and 3.09% for PenRed compared with egs_brachy. These values correspond to the two test cases that involved multi‐seeds in full‐tissue phantoms. Notably, the histogram peaks for these cases occur at the largest values of $\Delta D_{LOCAL}\left(\%\right)$ namely at values of −1.10% for MCNP and −3.00% for PenRed. Throughout all test cases, values of $\Delta D_{GLOBAL}\left(\%\right)$ remained within a narrow range, consistently centered around zero. This suggests that the systematic deviations were small relative to the prescription dose, indicating negligible impact when compared to egs_brachy. The absence of noticeable deviations in $\Delta D_{GLOBAL}\left(\%\right)$ confirms that the dosimetric results from all codes are consistent with one another and indicate negligible differences that would not affect clinical dose evaluation or decision‐making.

**FIGURE 3:**
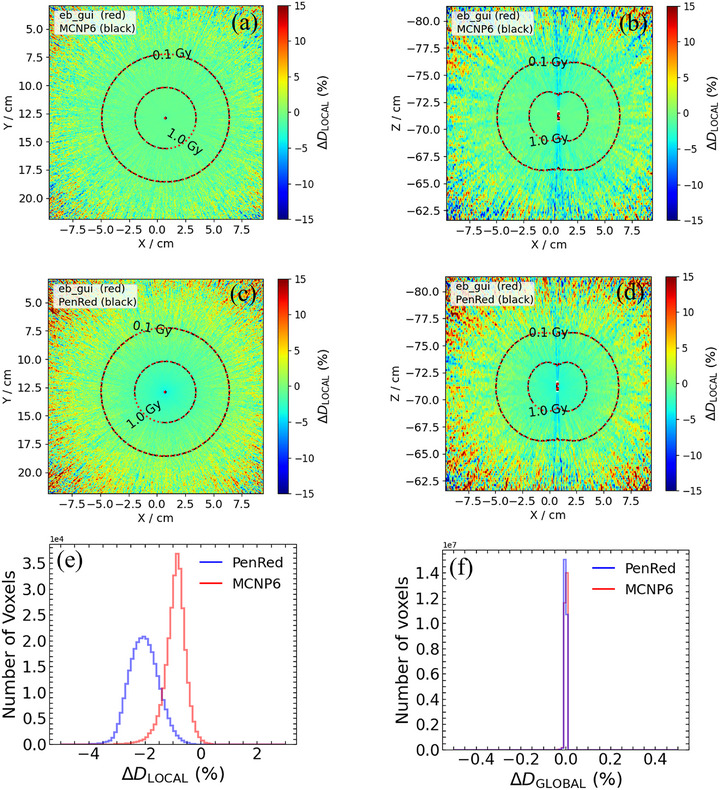
Results of comparison between codes for test case 1: Single seed in water (TG‐43). Local dose difference ratio, $\Delta D_{LOCAL}\left(\%\right)$, for MCNP (a) in plane *Z *= 0, (b) in plane *Y* = 0. $\Delta D_{LOCAL}\left(\%\right)$ for PenRed in (c) in plane *Z *= 0, (d) in plane *Y* = 0. Histogram of $\Delta D_{LOCAL}\left(\%\right)$ for voxels with doses greater than 1 Gy, and (e). Histogram of global dose difference ratios, $\Delta D_{GLOBAL}\left(\%\right)$, for the entire phantom geometry.

**FIGURE 4:**
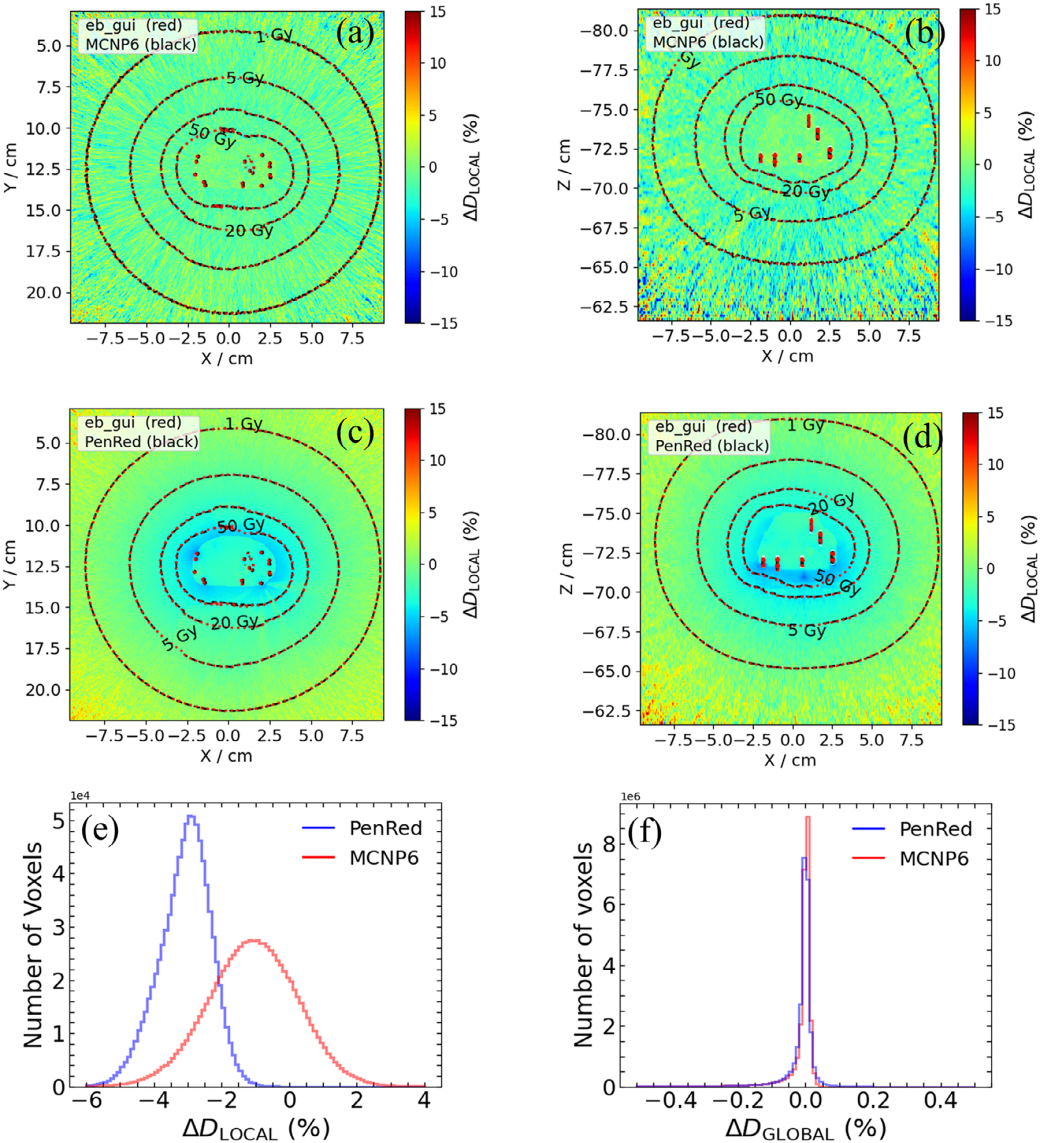
Results of comparison between codes for test case 6: Real virtual patient with calcifications, CT‐based full tissue phantom. Local dose difference ratio, $\Delta D_{LOCAL}\left(\%\right)$, for MCNP (a) in plane *Z* = 0, (b) in plane *Y* = 0. $\Delta D_{LOCAL}\left(\%\right)$ for PenRed in (c) in plane *Z* = 0, (d) in plane *Y* = 0. Histogram of $\Delta D_{LOCAL}\left(\%\right)$ for voxels with doses greater than 1 Gy, and (e). Histogram of global dose difference ratios, $\Delta D_{GLOBAL}\left(\%\right)$, for the entire phantom geometry.

Figures 3 and 4 display the colormaps of the local dose difference ratios for *Z* = 0 and *Y* = 0, along with histograms of $\Delta D_{LOCAL}\left(\%\right)$ and $\Delta D_{GLOBAL}\left(\%\right)$ for test case 1 and test case 6, respectively. The graphical analyses for other test cases are provided in the Supplementary Materials. These plots illustrate the overall dose discrepancies between the Monte Carlo codes. Both MCNP and PenRed generally yielded lower dose values compared to egs_brachy, as evidenced by the negative distribution of $\Delta D_{LOCAL}\left(\%\right)$. The largest discrepancies between codes were observed at the end caps of the seeds and along the long axis of the seeds (*Z*‐axis), where dose gradients are typically steeper. Additional differences were noted in the low‐dose regions, particularly those distant from the target.

#### 2.2.2 Dose‐Volume Histogram (DVH) comparisons

**FIGURE 5:**
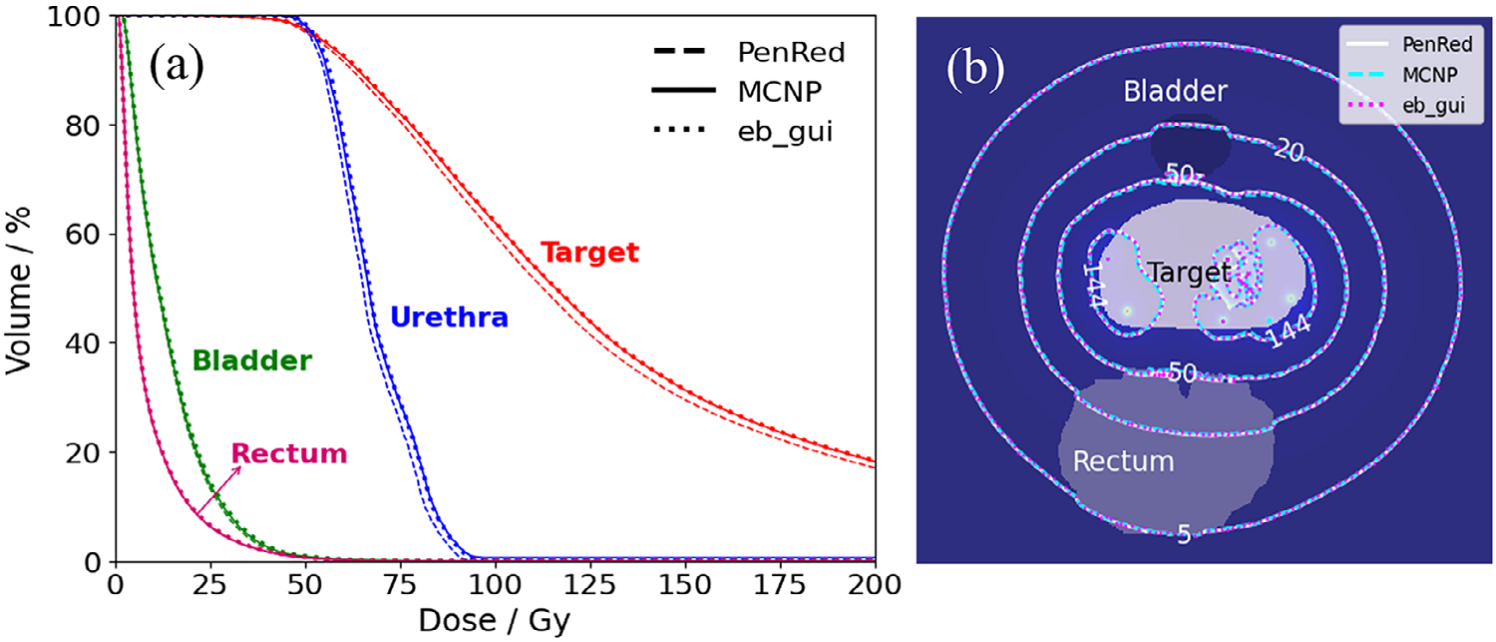
(a) Cumulative dose‐histogram (DVH) obtained with PenRed, MCNP, and eb_gui for the target, urethra, bladder, and rectum in test case 6. (b) Isodose lines obtained from three codes on central axial slice.

The cumulative dose‐volume histograms (DVHs) for the contoured structures in test case 6, obtained using three Monte Carlo codes are presented in Figure 5(a). The DVHs for the bladder and rectum show excellent agreement across all three codes, indicating consistent dose distributions for these organs. For the target and urethra, the DVHs exhibit a high degree of agreement between MCNP and eb_gui. However, a slight variation can be seen in the PenRed calculations, indicating minor differences in the predicted dose distribution compared to other codes. These discrepancies align well with the $\Delta D_{LOCAL}\left(\%\right)$ variations reported in previous sections, further supporting the observed differences in the DVH results. Figure 5(b) shows the isodose lines at 5, 20, 50, and 144 Gy, as calculated by the different codes. These isodose lines, overlaid with the segmented structures, demonstrate that the codes are in good agreement, with only minor variations in the predicted dose distributions.

## 3 DATA FORMAT AND USAGE NOTES

The prostate brachytherapy test cases are primarily available through the Zenodo repository, where all relevant files for reproducing the calculations can be downloaded. These test cases are also accessible via a link provided in the Brachytherapy Source Registry, a resource managed collaboratively by the AAPM and IROC Houston.^25^ All the files needed for reproducing these calculations can be downloaded and include:

- “DICOM data” containing 99 CT images of the prostate phantom, along with the RT structure set (RTSTRUCT) and RT plan (RTPLAN).
- 3D dose distributions for all MC codes and test cases, provided in .3ddose or .dcm format
- Input files for all MC codes for all test cases

Clinical users can adhere to the Level 2 MBDCA commissioning procedures outlined in TG‐186 and by AAPM Report 372^26^ by downloading and importing the provided DICOM files into their treatment planning system. They should then perform local MBDCA dose calculations for the test cases and compare their results with the reference MC results provided in this dataset. Furthermore, the dataset provides a useful benchmark for brachytherapy researchers requiring dosimetric assessments of various MBDCAs.

## 4 DISCUSSIONS

This work developed test cases for LDR prostate brachytherapy, covering scenarios from a single seed placed in a homogeneous water medium to complex configurations involving multiple seeds embedded in a CT‐based, full‐tissue phantom, that also includes calcifications to represent realistic patient conditions. The described test cases offer a structured framework for evaluating various model‐based dose calculation algorithms used in brachytherapy and facilitate their commissioning for clinical use. The workflow outlined in the AAPM TG‐186 report served as the foundation for these test cases, ensuring alignment with best practices for clinical implementation.

In this study, eb_gui was designated as the reference code due to its comparatively lower Type A uncertainties compared to other codes. It is important to note that MCNP and PenRed used phase space sources which allow the codes to start particle simulations from a pre‐defined, detailed distribution. This approach can significantly reduce the number of histories required to achieve statistical accuracy, leading to shorter computational times.

Test cases 1, 3, and 4 focused on single or multiple seeds placed within a homogenous water medium. In test case 1, which involved a single seed in water, the local dose differences between the MC codes observed in this work remained well within the Type A uncertainties, demonstrating a high degree of agreement among the codes. These findings are consistent with a previous study conducted by WGMBDCA,^24^ in which test cases were created for eye plaque brachytherapy using a similar fully modeled I‐125 seed and a comparable photon emission spectrum. In that study, three MC codes—egs_brachy, MCNP, and Penelope—were used for simulations. Our results show better agreement in the $\Delta D_{LOCAL}\left(\%\right)$ between eb_gui and MCNP, while a slightly poorer agreement was observed between eb_gui and PenRed. Additionally, in our simulations, the distributions of $\Delta D_{LOCAL}\left(\%\right)$ for both codes are consistently negative. However, they include regions with values both higher and lower than those of egs_brachy. These differences can be attributed to the slightly different MC parameters used in the two studies. As illustrated in Figures S2 and S3 in the Supplementary Materials, the local dose difference ratios between MCNP and eb_gui for test cases 3 and test 4, both involving multiple seeds in water, were normally distributed, with means of −0.36 ± 1.17% and −0.88% ± 1.35%, respectively. These results show that the local dose differences were, on average, minor, with a systematic deviation of less than ∼1%. The mean local difference ratios between PenRed and eb_gui were −1.67 ± 0.73% for test case 3 and −2.80% ± 0.81% for test case 4. A deviation of −1.1% was noted for test case 3, which increased to −2.4% in test case 4. Despite these differences, all values remained within Type A uncertainties, further confirming that, for simpler scenarios with seeds in homogeneous media, the MC codes perform similarly. This agreement is critical for ensuring reliable dose calculations in clinical brachytherapy.

However, when transitioning to more complex and realistic test cases involving a full‐tissue prostate phantom (test cases 2, 5, and 6), the deviations between the MC codes became more pronounced. For MCNP, the dose deviations remained relatively modest, ranged from −0.9% to −1.1%. In contrast, PenRed exhibited larger deviations, ranging from −2.1% to −3.1%. These differences may be attributed to variations in how each code models and assigns material properties, particularly with respect to tissue heterogeneities and boundary definitions. Additionally, intrinsic differences in the implementation of mass‐energy absorption coefficients are likely contributors to the observed discrepancies. A conservative Type B uncertainty estimate (*k* = 2) for the absorbed dose in low‐energy photon range (<50 keV) has been reported to lie between 1.2% and 1.7%.^50^ In PenRed, the mass‐energy absorption coefficients are derived from renormalized photoelectric cross sections that incorporate multiconfiguration Dirac‐Fock effects.^47^, ^51^ This renormalization, based on Pratt's screening approximation, improves the treatment of atomic structure effects that are not captured in simpler models. In contrast, eb_gui and MCNP utilize unnormalized photoelectric cross sections which may partly explain the lower deviation observed for MCNP relative to eb_gui compared to PenRed in these test scenarios. A comparison of the mass‐energy absorption coefficients used in different codes is shown in Figure S5 in the Supplementary Materials, showing differences of about 5% in the 5–30 keV energy range.

The larger range of local difference ratios observed in the full‐tissue phantom test cases can also be attributed to lower‐dose regions in the phantom, such as areas outside the clinical target volume. These regions, which typically receive less radiation, exhibit more variability due to the spatial distribution of dose. To address this, applying dose thresholds could narrow the histogram, focusing the analysis on more clinically relevant regions.

Another major contributing element to the observed disparities is the way that material assignments are handled in the different MC codes. Notably, eb_gui employs a contour priority approach, which prioritizes certain anatomical structures, such as the urethra, when assigning materials to overlapping regions. This approach can lead to differences in voxel assignments when the urethra overlaps with other structures, such as calcifications. For instance, voxels that contain both calcifications and the urethra are allocated to the urethra in eb_gui, but they may be assigned to the calcifications in other MC codes. This discrepancy can result in visible differences in the dose distribution. Another important source of disagreement between the MC codes is the shadowing effect resulting from photon attenuation in areas behind calcifications. This effect is more noticeable in regions with dense materials, such as calcifications, which can block or scatter photons, thereby reducing the dose delivered to adjacent tissues. The differences in how these shadowing effects are modeled, combined with inherent variations in the mass‐energy absorption coefficients used by each code, contribute to the observed discrepancies (similar to observations elsewhere^52^).

While commercially available MBDCAs for permanent LDR prostate implants remain limited and full Monte Carlo implementations can be computationally demanding for routine clinical use, the Monte Carlo–based reference datasets generated in this study provide a high‐accuracy, reproducible framework for benchmarking and validating algorithms, supporting development, optimization, and future clinically feasible implementation.

## 5 CONCLUSIONS

In this study, we developed a diverse set of six test cases, ranging from a basic single‐seed model in water to a detailed prostate phantom, to enable thorough evaluation and resolution of discrepancies between different model‐based dose calculations. This framework can be extended to other low‐energy brachytherapy applications by creating similar test cases. The Monte Carlo codes showed an excellent overall agreement. We provide 3D dose distributions and phantoms to support researchers and clinicians in benchmarking model‐based dose calculation algorithms.

## CONFLICT OF INTEREST STATEMENT

The authors declare that they do not have any pertinent conflicts of interest.

## Supporting information



Supporting information

## Data Availability

The test cases developed in this study are available at https://doi.org/10.5281/zenodo.15282647.
