# Supplementary material for: Generation of 3D reference dosimetric datasets towards adoption of model‐based dose calculations for permanent implant prostate brachytherapy
Source: Med Phys. 2026 Feb 10;53(2):e70309. doi: 10.1002/mp.70309 (PMC12888956; doi:10.1002/mp.70309)
Supplement: Supplementary file 1 — Supporting information [file MP-53-0-s001.pdf]

Supplementary Materials for:

Generation of 3D reference dosimetric datasets towards adoption of model-based dose calculations for permanent implant prostate brachytherapy

Akbari F, Peppa V, Ouellet S, et al. Generation of 3D reference dosimetric datasets towards adoption of model-based dose calculations for permanent implant prostate brachytherapy. *Med Phys*. 2026;e70309. https://doi.org/10.1002/mp.70309

This document provides a broader range of graphical analyses to supplement the findings in the original study. For each test case, it includes colormaps displaying both local and global percentage dose differences in different planes, as well as histograms showing these dose differences. A summary of the test cases and figures is provided in Table S1.

The test cases developed in this study are available at http://doi.org/10.5281/zenodo.15282647.

Table S1. Overview of the studied test cases

Test case number	Description	Seed configuration	Phantom	Figure number
2	Single seed in phantom	Single seed	CT-based full tissue phantom	1
3	Multiseed, no interseed effects (TG-43)	58 seeds	Water	2
4	Multiseed, interseed effects	58 seeds	Water	3
5	Real virtual patient without calcifications	58 seeds	CT-based full tissue phantom, without calcifications	4

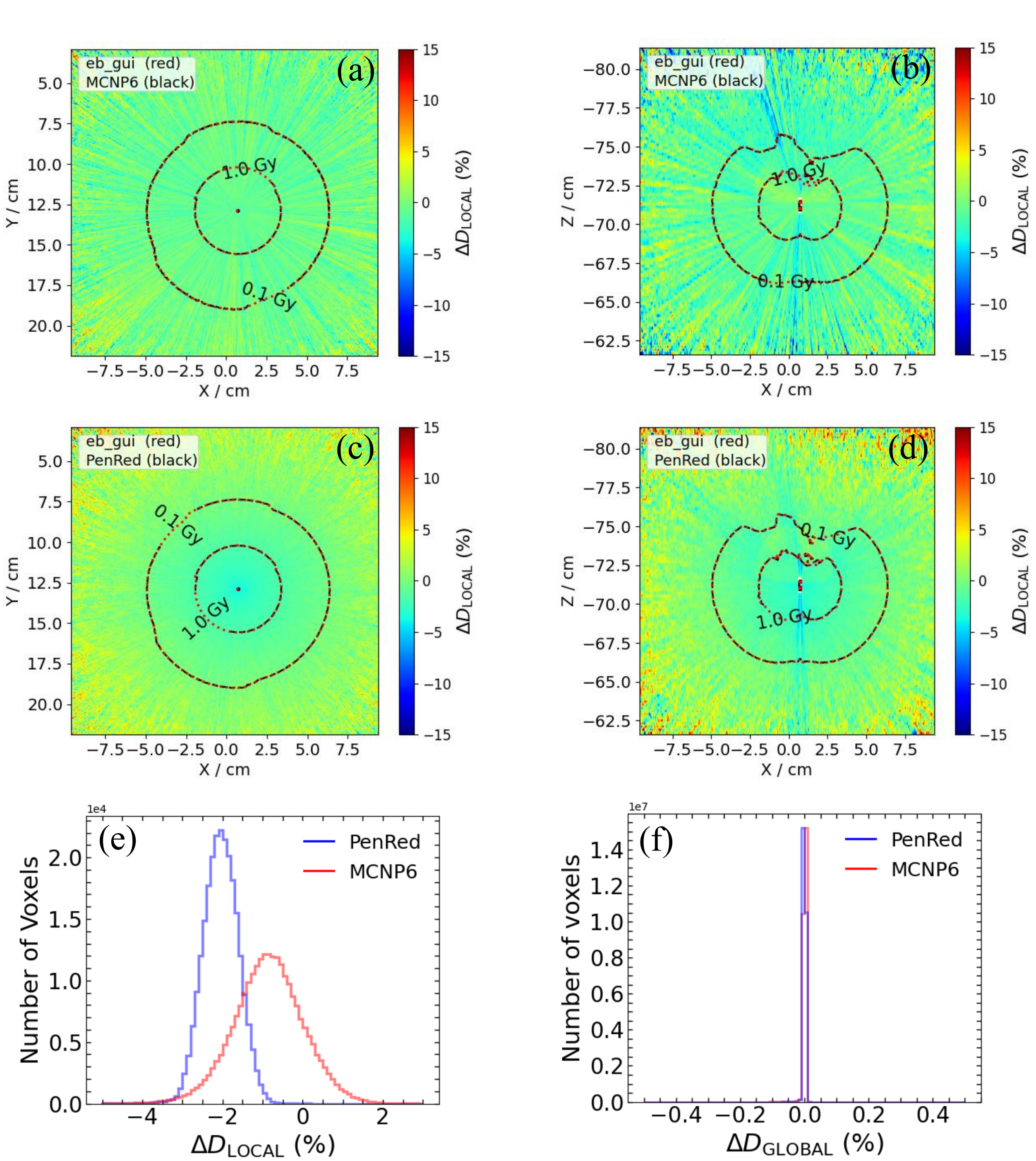

Figure S1. Results of comparison between codes for test case 2: Single seed in CT-based full tissue phantom. Local dose difference ratio for MCNP (a) in plane Z= 0, (b) in plane Y= 0. Local dose difference ratio for PenRed in (c) in plane Z= 0, (d) in plane Y= 0. Histogram of local dose difference ratios for voxels with doses greater than 1 Gy, and (e). Histogram of global dose difference ratios for the entire phantom geometry. The irregularities observed in the isodose lines in Figure S1(b) and (d) arise from heterogeneities in the CT-based phantom.

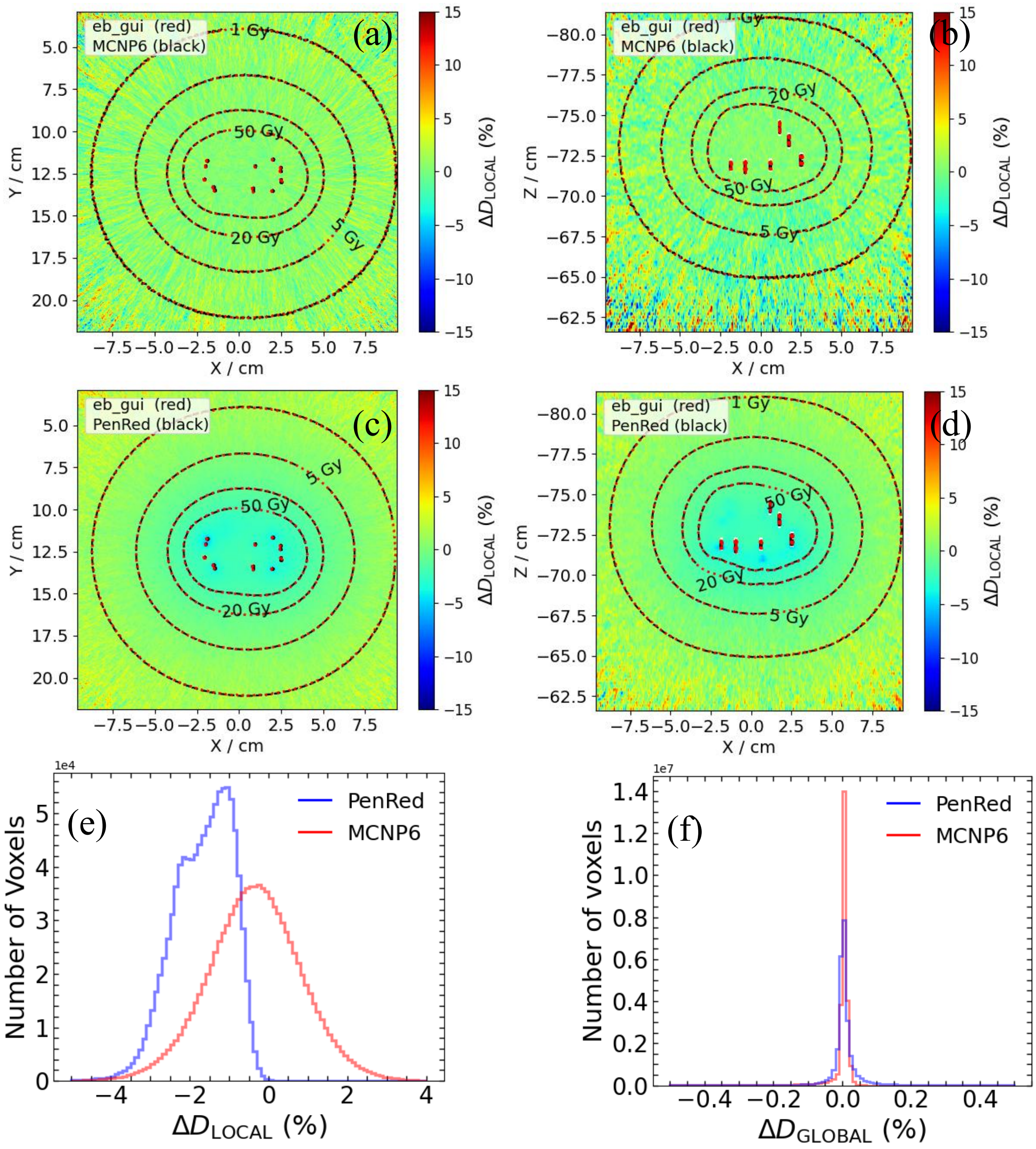

Figure S2. Results of comparison between codes for test case 3: Multiseed, no interseed effects (TG-43). Local dose difference ratio for MCNP (a) in plane Z= 0, (b) in plane Y= 0. Local dose difference ratio for PenRed in (c) in plane Z= 0, (d) in plane Y= 0. Histogram of local dose difference ratios for voxels with doses greater than 20 Gy, and (e). Histogram of global dose difference ratios for the entire phantom geometry.

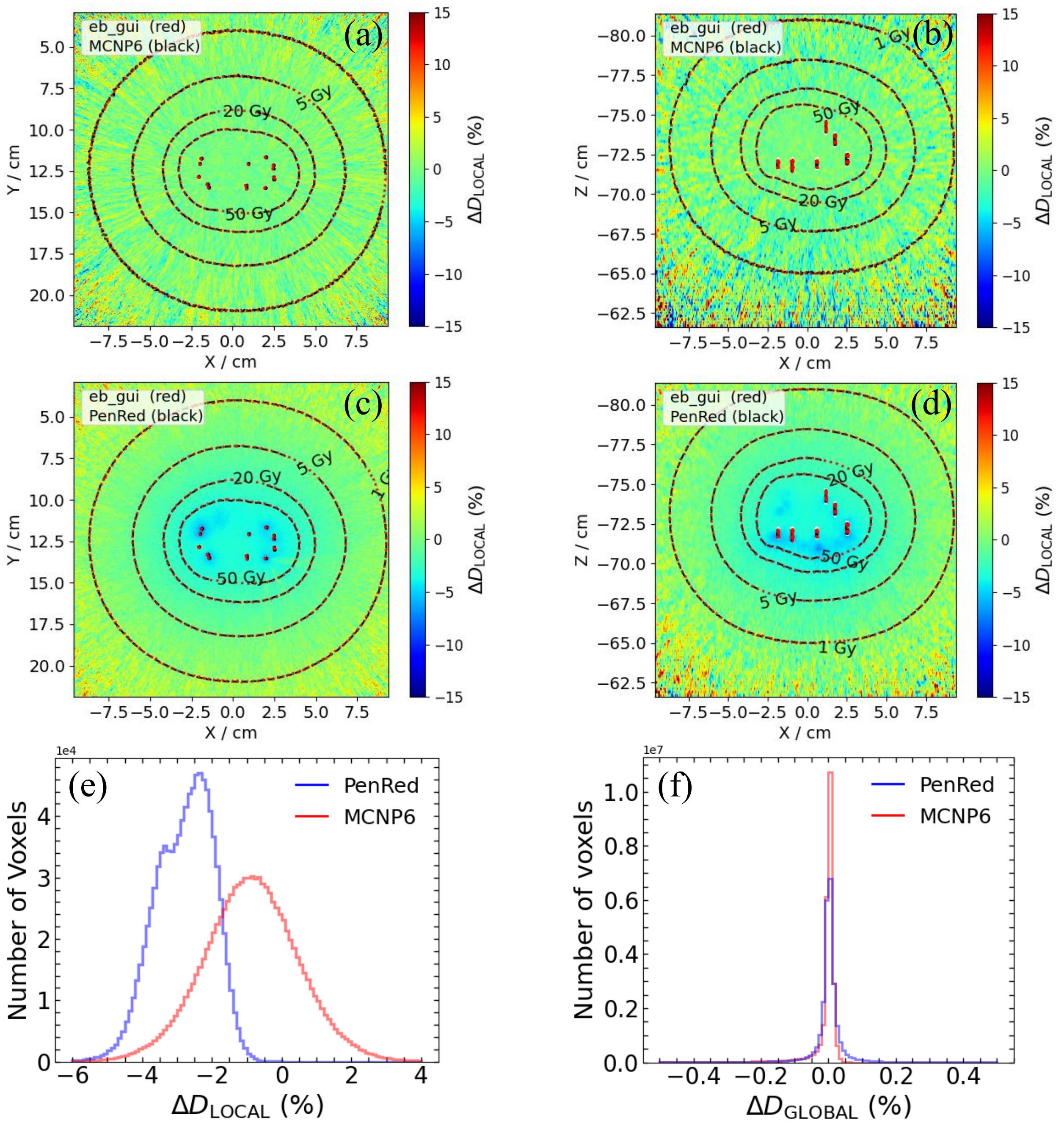

Figure S3. Results of comparison between codes for test case 4: Multiseed, with interseed effects. Local dose difference ratio for MCNP (a) in plane Z= 0, (b) in plane Y= 0. Local dose difference ratio for PenRed in (c) in plane Z= 0, (d) in plane Y= 0. Histogram of local dose difference ratios for voxels with doses greater than 20 Gy, and (e). Histogram of global dose difference ratios for the entire phantom geometry.

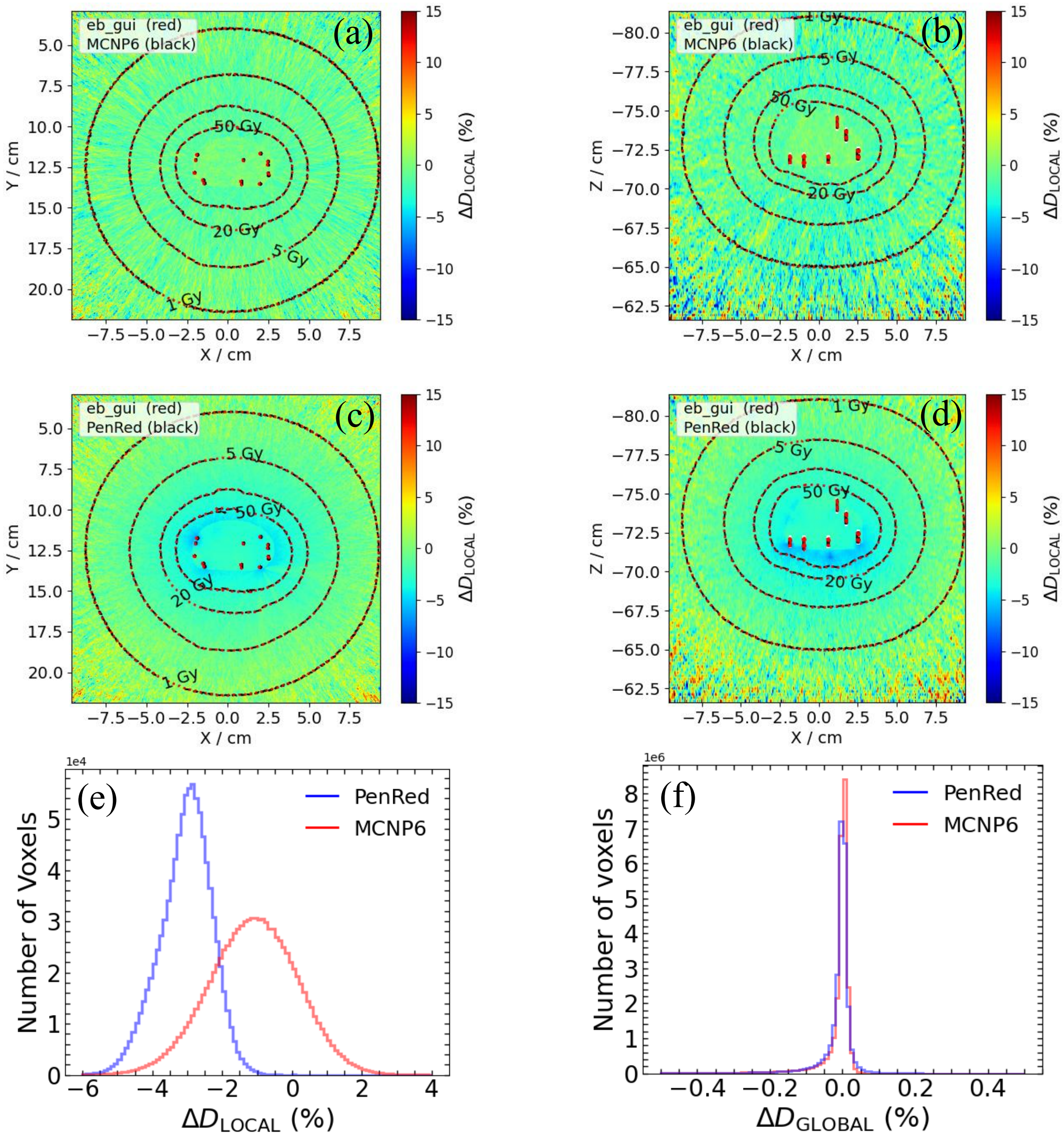

Figure S4. Results of comparison between codes for test case 5: Real virtual patient without calcifications, CT-based full tissue phantom, without calcifications. Local dose difference ratio for MCNP (a) in plane Z= 0, (b) in plane Y= 0. Local dose difference ratio for PenRed in (c) in plane Z= 0, (d) in plane Y= 0. Histogram of local dose difference ratios for voxels with doses greater than 20 Gy, and (e). Histogram of global dose difference ratios for the entire phantom geometry.

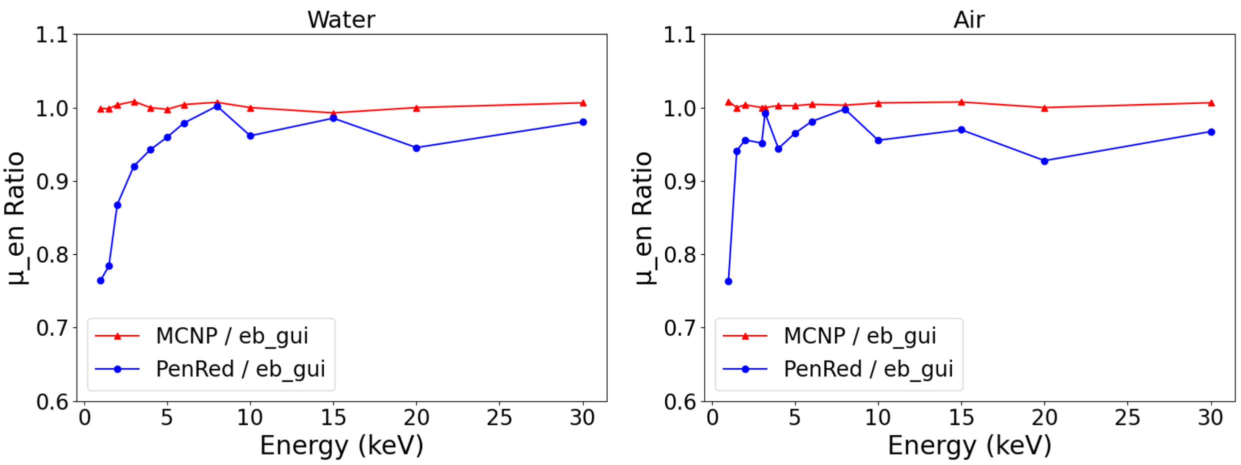

Figure S5. Comparison of mass-energy absorption coefficients for water and air used in different codes. The plot shows the ratio of mass-energy absorption coefficients from each code to that of eb_gui.
